# Skill Learning Modulates RNA Pol II Poising at Immediate Early Genes in the Adult Striatum

**DOI:** 10.1523/ENEURO.0074-17.2017

**Published:** 2017-04-17

**Authors:** Pedro Galvão-Ferreira, Michal Lipinski, Fernando Santos, Angel Barco, Rui M. Costa

**Affiliations:** 1 Champalimaud Neuroscience Programme, Fundação Champalimaud, Lisbon, 1400-038 Portugal; 2 Instituto de Neurociencias, Universidad Miguel Hernández - Consejo Superior de Investigaciones Científicas, Alicante, 03550 Spain

**Keywords:** learning, motor skill, RNA Pol II, RPB1, striatum

## Abstract

A multilayered complexity of epigenetic and transcriptional regulatory mechanisms underlies neuronal activity-dependent gene transcription. The regulation of RNA Pol II progression along the transcription cycle, from promoter-proximal poising (with RNA Pol II paused at promoter-proximal regions, characterized by a Ser5P^+^-rich and Ser2P^+^-poor RPB1 CTD) to active elongation, has emerged as a major step in transcriptional regulation across several organisms, tissues, and developmental stages, including the nervous system. However, it is not known whether this mechanism is modulated by experience. We investigated the impact of learning a motor skill on RNA Pol II phosphorylation dynamics in the adult mouse striatum. We uncovered that learning modulates the *in vivo* striatal phosphorylation dynamics of the CTD of the RNA Pol II RPB1 subunit, leading to an increased poising index in trained mice. We found that this modulation occurs at immediate early genes (IEGs), with increased poising of RNA Pol II at both *Arc* and *Fos* genes but not at constitutively expressed genes. Furthermore, we confirmed that this was learning dependent, and not just regulated by context or motor activity. These experiments demonstrate a novel phenomenon of learning induced transcriptional modulation in adult brain, which may have implications for our understanding of learning, memory allocation, and consolidation.

## Significance Statement

RNA Pol II poising is a powerful way of modulating gene transcription. Although previous studies have shown activity-dependent changes in RNA Pol II poising *in vitro*, the modulation of RNA Pol II poising by experience has not been investigated. In this study, we show that learning modulates striatal phosphorylation dynamics of the RNA Pol II RPB1 subunit *in vivo*, leading to an increased poising index in trained mice. We also show that learning modulates RPB1 phosphorylation at immediate early genes (IEGs), with increased poising of RNA Pol II in both *Arc* and *Fos* genes. Our experiments demonstrate a new phenomenon of learning-induced transcriptional modulation in the adult brain that may be involved in neural circuit-priming, memory consolidation and recall.

## Introduction

The nervous system mediates the interactions between animals and the environment. These interactions are modified through changes in neuronal connectivity, neuronal structure, and neuronal activity that mold neural circuits in an experience-dependent manner (Lyons and West, 2011; West and Greenberg, 2011). Skills are learned gradually, but once they are, they can last a lifetime (Shadmehr and Brashers-Krug, 1997; Karni et al., 1998). Long-lasting consolidation of skills requires neuronal adaptability in different brain systems at different levels, and it may include adjustments to the transcription of neuronal genomes. The striatum, the entry gateway to the basal ganglia, and corticostriatal plasticity have been implicated in skill learning (Barnes et al., 2005; Yin et al., 2009; Jin and Costa, 2010; Jin et al., 2014; Santos et al., 2015). Although the neuronal circuits responsible for striatal-dependent instrumental learning have been identified, the molecular mechanisms behind long-lasting skill consolidation are less understood.

Chromatin remodeling and transcriptional regulation are critical for experience-dependent gene expression (Lyons and West, 2011; West and Greenberg, 2011; Benito and Barco, 2015). By packing the genetic information contained in genomes and regulating its transcription, chromatin bridges the structural accessibility of genes into spatially regulated nuclear gene expression (Hager et al., 2009; Levine et al., 2014). Many epigenetic mechanisms, from acetylation and methylation of histones to cytosine DNA methylation, have a comprehensive impact on gene expression as they help orchestrate a harmonious sequence of chromatin remodeling and effective transcriptional regulation (Wolf and Linden, 2012). Many of these epigenetic regulatory mechanisms mediate neuroplasticity by linking the activity of chromatin remodeling enzymes (such as histone deacetylases) to Ca^2+^-dependent signaling proteins and activity-dependent transcription factors (Hager et al., 2009; Meaney and Ferguson-smith, 2010; Wolf and Linden, 2012; Levine et al., 2014; Lopez-Atalaya and Barco, 2014).

Transcription itself may be regulated at multiple stages. One of the possible checkpoints is the progression of RNA Pol II throughout the transcription cycle by phosphorylation of the serine residues along the heptapeptide consensus sequence Tyr-Ser-Pro-Thr-Ser-Pro-Ser (Y_1_S_2_P_3_T_4_S_5_P_6_S_7_) at the C-terminal domain (CTD) of its largest subunit, RPB1 (Jonkers and Lis, 2015). RNA Pol II transcriptional progression rests on a balance between an enrichment of RNA Pol II RPB1 phosphorylated at Ser5 (Ser5P^+^) close to the transcription start site, and an increase of Ser2 phosphorylated RPB1 (Ser2P^+^) in actively transcribing RNA Pol II (Jonkers and Lis, 2015). First identified in *Drosophila melanogaster* heat shock protein (*hsp*) genes (Gilmour and Lis, 1986; Rougvie and Lis, 1988, 1990; Rasmussen and Lis, 1993), this ability of RNA Pol II to pause in promoter-proximal regions is also present in neurons of the central nervous system, where it has been shown to regulate the activity-dependent transcriptional dynamics of immediate early genes (IEGs; Saha et al., 2011). However, this mechanism has not been studied in the adult brain *in vivo* in the context of learning. With this in mind, we set out to explore the impact of learning a motor skill on RNA Pol II poising in the mouse striatum. Using a fast lever-pressing task as a motor skill-learning paradigm, we examined the global phosphorylation dynamics of RNA Pol II in adult mouse striatum, and subsequently profiled RPB1 phospho-variant binding to the promoters and gene bodies of the IEGs *Arc* and *Fos*. We report modulation of RPB1 CTD phosphorylation at IEGs in response to learning, resulting in a dynamically changing Ser5P^+^/Ser2P^+^ ratio (the poising index). These experiments demonstrate a novel instance of learning-induced transcriptional modulation via RNA Pol II phosphorylation in the brain.

## Materials and Methods

### Animals

All procedures were reviewed and performed in accordance with the Champalimaud Center for the Unknown Ethics Committee guidelines, and approved by the Portuguese Veterinary General Board (Direcção Geral de Veterinária, approval 0421/000/000/2014). All animals used in the present study were male C57BL/6J mice between two and five months of age kept on a 12-h light/dark cycle. Experiments were performed on the light cycle.

### Behavioral procedures

Behavioral training took place in operant chambers (21.6 cm L × 17.8 cm W × 12.7 cm H) housed within sound attenuating chambers (MedAssociates). Each chamber was equipped with two retractable levers on either side of the food magazine and a house light (3 W, 24 V) mounted on the opposite side of the chamber. Reinforcers were delivered into the magazine through a pellet dispenser, and magazine entries were registered using an infrared beam. Before training started, mice were placed on a food deprivation schedule, receiving 1.5–2 g of food per day, allowing them to maintain a body weight above 85% of their baseline weight. Throughout training, mice were fed daily after the training session. Mice were trained with 20-mg “chow” pellets (Bio-Serv) as reinforcers, with the delivery of these in the operant chamber contingent on lever pressing. Training started with a 60-min magazine training session in which one reinforcer was delivered on a random time schedule on average every 2 min (30 reinforcers). The following day, lever-pressing training started, with each animal learning to press the lever to obtain a reinforcer. Each daily session started with the illumination of the house light and insertion of the lever, and ended with the retraction of the lever and the offset of the house light; sessions lasted for 60 min or until animals received a total of 30 reinforcers, with one training session per day. In the first training session, animals were subjected to continuous reinforcement with each lever-press leading to the delivery of one reinforcer into the magazine (to a maximum of 30 reinforcers; CRF30). After CRF, animals were trained in a fixed ratio (FR) schedule, in which delivery of a reinforcer resulted from eight lever presses (FR8) within a time contingency, resulting in a minimum frequency (covert target): FR8-1000s (i.e., eight lever presses within 1000 s); FR8-500s; FR8-50s; FR8-10s; FR8-5s; FR8-4s; FR8-3s; FR8-2s; FR8-1s, with animals finishing their fast lever-pressing training at 8 Hz. This constant increase in the minimum frequency of the covert target forced the animals to systematically adapt to the task requirements and perform faster sequences of presses from session to session. Animals were trained in the fast lever-pressing task, and a control group (context control animals) was simultaneously exposed to behavioral operant chambers without performing any operant lever-pressing task and hence not receiving the corresponding reinforcers (this being the control group present in all figures, unless otherwise stated). Two additional control groups of animals were run: a group in which in addition to being exposed to behavioral boxes, animals were fed a maximum of 30 reinforcers per exposure session (dubbed “reinforcement control” animals), similar to the experimental subjects on completion of fast lever-pressing task sessions; and a control group of “performance control” animals, where mice were trained in the fast lever-pressing task and killed after completion of FR8-50s (to roughly correspond to a halfway point in the training regime).

In the experiments of Figure 5, trained animals and performance controls were pooled, and divided into two groups based on their performance (number of presses below or above 250 presses) of their learning of the skill (proximity to target below or above 0.6).

#### Sequences of lever presses

Sequences of lever presses were differentiated based on interpress interval (IPI) and occurrence of a magazine head entry. An IPI > 2 s (determined based on the distribution of IPIs) or a head entry were used to define the bouts or sequences of presses.

### Western blotting

To dissect whole striata, mice were anesthetized immediately after the termination of behavioral experiments using a mix of oxygen (1–1.5 l/min) and isoflurane (1–3%), killed by cervical dislocation, their brains quickly removed and transferred to ice-cold PBS. Total striatum was dissected from both hemispheres, flash-frozen in liquid nitrogen and kept at -80°C until used. Total protein was extracted from the pooled bilateral striata of each mouse by lysis of tissue samples in 400 μl of ice-cold RIPA buffer (Sigma-Aldrich, #R0278) supplemented with phosphatase and protease inhibitors (PhosSTOP Roche #04906837001, and Complete Tablets EDTA-free Roche 04693159001, respectively), homogenization using 1.5-ml microcentrifuge tube-adaptable disposable tissue grinder pestles (Capitol Scientific, #199230000), disruption by brief sonication and pipetting up and down twenty times with a P200 pipette tip. Protein concentration was assayed using the Pierce BCA Protein Assay kit (Thermo Scientific #23227) with the absorbance measured at 562 nm on a plate reader, with each animal yielding a protein concentration of 3000-4000 μg/ml. One part of 4× Laemmli sample buffer (Bio-Rad #161-0747), containing 2-Mercaptoethanol (Bio-Rad #161-0710) in a 1:10 dilution, was added to three parts of protein sample (∼40 μg of protein per well), boiled at 95°C for 5 min, and resolved in 4–15% gradient precast SDS-PAGE gels (Mini-PROTEAN TGX Stain-Free Gels, 10 well, Bio-Rad #456-8083) in 1× running buffer (diluted 1:5 from a 5× stock: 0.125 M Tris base, 1 M glycine, 0.017 M SDS), together with a protein ladder for reference (Bio-Rad 1× Precision Plus Protein WesternC Standards, #161-0376) at 100V for ∼1.5 h. Proteins were semi-dry transferred to PVDF membranes (Bio-Rad #162-0177) for 1 h at 12 V in 1× transfer buffer (diluted 1:5 from a 5× stock: 0.125 M Tris Base, 0.96 M glycine). PVDF membranes were then blocked in 5% Blotting-Grade Blocker (Bio-Rad #170-6404) in TBS-0.1%Tween 20 (TBS: 0.1 M Tris and 1.5 M NaCl, pH at 7.4) for 1 h at room temperature (RT). After blocking, PVDF membranes were incubated with the primary antibody at a 1:500 dilution, as well as with an antiactin antibody (Sigma #A5441) at a 1:200,000 dilution, in TBS-0.1%Tween 20 with 5% Blotting-Grade Blocker overnight at 4°C. Anti-RPB1 primary antibodies used: total RPB1 subunit, clone H224 (Santa Cruz Biotechnology #SC-9001X); Ser5P^+^ RPB1 CTD, clone CTD4H8 (Millipore/Millipore #05-623); Ser2P^+^ RPB1 CTD, clone H5 (Covance #MMS-129R; Stock et al., 2007). After primary antibody incubation, membranes were rinsed three times for 5 min with TBS-0.1%Tween 20 at RT and incubated with the HRP-conjugated secondary antibody at a 1:2000 dilution in TBS-0.1%Tween 20 with 5% Blotting-Grade Blocker for 1 h at RT. Secondary antibodies used: anti-mouse (Dako #P0260); anti-goat (Invitrogen #G21234). Membranes were then once again washed three times for 5 min with TBS-0.1%Tween 20 at RT. The chemiluminescent substrate (Clarity Western ECL Substrate, Bio-Rad #170-5060) was added to the blot for 5 min at RT according to the manufacturer’s recommendations. Chemiluminescent signals were detected in an automated chemiluminescence imager for protein high-resolution digital imaging (GE Healthcare Imager 600). Protein bands were quantified using ImageJ software, with Total RPB1 subunit, Ser5P^+^ RPB1 CTD and Ser2P^+^ RPB1 CTD signals normalized to actin in the respective well.

### Chromatin immunoprecipitation (ChIP) followed by Real-Time PCR (qPCR)

Similar to Western blot analysis, mice were anesthetized immediately after the termination of behavioral experiments using a mix of oxygen (1–1.5 l/min) and isoflurane (1–3%), killed by cervical dislocation, their brains quickly removed and transferred to ice-cold PBS. Total striatum was dissected from both hemispheres, flash-frozen in liquid nitrogen and kept at −80°C until used.

#### Preparation of Dynabeads protein G

Dynabeads (Life technologies-Invitrogen-Novex 10004D) were mixed well and aliquoted (60 μl per immunoprecipitation reaction), and one tube per antibody prepared. One ml of cold PBS was added to the beads, gently vortexed to mix and the tube placed in a magnetic stand. Tubes were inverted several times to mix, and beads were allowed to clump for ∼1 min. PBS was pipetted off, and this wash step repeated two more times. The specific antibodies were added to the beads: total RPB1 subunit, clone H224 (Santa Cruz Biotechnology #SC-9001X) 5 μg/reaction; anti-RNA polymerase II Ser2P^+^ RPB1 CTD repeat YSPTSPS antibody, ChIP grade: ab5095, 8 μg/reaction; anti-RNA polymerase II Ser5P^+^ RPB1 CTD repeat YSPTSPS antibody, ChIP grade: ab5131, 3 μg/reaction (Hoogenkamp et al., 2007; Stock et al., 2007; Hargreaves et al., 2009). The volume was adjusted to 1.5 ml with RIPA-150 buffer (50 mM Tris-HCl, pH 8.1, 150 mM NaCl, 1 mM, EDTA, pH 8, 0.1% SDS, 1% Triton X-100, and 0.1% sodium deoxycholate), and antibodies were prebound for at least 5 h at 4°C on an orbital rotator. While beads were incubated with the antibody, the following cross-linking and lysis steps were performed.

#### In vivo cross-linking and lysis

Tubes (1.5 ml) were prepared containing 940-μl PBS and 60-μl fresh formaldehyde (FA) 18.5%, with one tube per mouse bilateral striata. Tissue was chopped using a single-edge razor, transferred into the previously prepared 1.5-ml tube with FA solution and incubated at RT for 10 min in an orbital rotator. 110 μl of 1.25 M glycine were then added and incubated at RT for 5 min to quench unreacted FA. Tubes were spinned at 700*g* for 3 min to pellet tissue and the PBS/FA/glycine solution was aspirated. The tissue was then washed with 1 ml of PBS. The previous 700*g* spin and 1-ml PBS wash cycle was repeated three times, to a total of three washes. Next, 500 μl of lysis buffer N (50 mM HEPES-KOH, pH 8.1, 1 mM EDTA, 0.5 mM EGTA, 140 mM NaCl, 10% glycerol, 0.5% NP-40, 0.25% Triton X-100) with protein inhibitor mixture (Roche #04693159001) were then added to the pellet and homogenized using a Heidolph Diax 900 homogenizer at level 1 for 10-20 s or until no clumps were present in the solution. The homogenate (500 μl) was placed into a 15-ml tube containing 10 ml of lysis buffer N with protein inhibitor mixture, incubated at 4°C for 10 min with orbital rotation and then spinned at 600*g* for 5 min at 4°C to pellet nuclei. Nuclei were washed with 10 ml of wash buffer N (10 mM Tris-HCl, pH 8.0, 1 mM EDTA, 0.5 mM EGTA, 200 mM NaCl) at 4°C for 10 min with orbital rotation, and pelleted again (600*g* for 5 min at 4°C). The supernatant was aspirated and pelleted nuclei resuspended in 100 μl of SDS lysis buffer (1% SDS, 10 mM EDTA, 50 mM Tris, pH 8.1). Samples were transferred to 0.5-ml LoBind Eppendorf microcentrifuge tubes and sonicated in a Bioruptor (Diagenode) for 20 cycles (30 s on/30 s off). Samples were then centrifuged for 6 min at 13,000 rpm at RT. The pellet (containing insoluble particles) was discarded, and the supernatant (containing sheared chromatin) was transferred to new 1.5-ml LoBind tubes. Five microliters (5%, for the total RPB1 subunit experiment) or 10 μl (10%, in the Ser2P^+^ and Ser5P^+^ RPB1 experiments) of sheared chromatin were set aside to evaluate shearing efficiency and to measure chromatin concentration (by adding 200 μl of freshly made direct elution buffer (10 mM Tris-HCl, pH 8, 300 mM NaCl, 5 mM EDTA, pH 8, 0.5% SDS) and performing the protein/DNA complex elution and reverse cross-linking to ethanol precipitation steps described below; then dissolving each of the precipitated DNA samples in 20 μl of 10 mM Tris-Cl, pH 8.1, using 5 μl to quantify DNA in a Nanodrop system and 15 μl to run in a 1.2–1.5% agarose gel (corresponding to 3% of the whole chromatin sample per sample); DNA fragment size should be in the range of 200-800 bp.

#### Immunoprecipitation of cross-linked protein/DNA

The antibody-bound Dynabeads prepared above were placed in a magnetic stand and inverted several times. Beads were then allowed to clump and the supernatant discarded, with beads being kept on ice. Sonicated chromatin was diluted 1:10 in ChIP dilution buffer (0.01% SDS, 1.1% Triton X-100, 1.2 mM EDTA, 16.7 mM Tris-HCl, pH 8.1, 167 mM NaCl) with protein inhibitor mixture (the final volume should be 1 ml). A total of 1% (10 μl) of the supernatant was removed as input and saved at 4°C (or −20°C). Diluted chromatin was added to antibody-bound Dynabeads, gently mixed and placed on a rocker O/N at 4°C. Tubes were then placed in a magnetic stand and inverted several times. Beads were allowed to clump and the supernatant was discarded. The Dynabeads protein G-antibody/chromatin complexes were washed by resuspending the beads in 1 ml each of the cold buffers: RIPA-150 buffer for two washes; RIPA-500 buffer (50mM Tris-HCl, pH 8.1, 500 mM NaCl, 1 mM, EDTA, pH 8, 0.1% SDS, 1% Triton X-100, 0.1% sodium deoxycholate) for three washes; RIPA LiCl buffer (50 mM Tris-HCl, pH 8.1, 1 mM EDTA, pH 8, 1% NP-40, 0.7%, sodium deosycholate, 500 mM LiCl_2_) for two washes; TE buffer (10 mM Tris-HCl, pH 8.0, 1 mM EDTA, pH 8.0) for two washes; suds were aspirated after final wash) and incubated for 5 min on a rocker at 4°C.

#### Elution of protein/DNA complexes and reversal of protein/DNA complex cross-linking

Beads were resuspended in 200 μl of freshly made direct elution buffer (with 200 μl of freshly made direct elution buffer also added to input samples). From this point on, the protocol was conducted with proper samples and the saved 1% input samples. A total of 1-μl RNase A 10 mg/ml (Fermentas #EN0531) was added and incubated for 6 h to O/N at 65°C to reverse cross-link (samples were kept at 1000 rpm in a termoblock to keep them in suspension). Samples were then quickly spinned and placed on a magnetic stand, allowing beads to clump and supernatants transferred to new LoBind tubes. A total of 3 μl of proteinase K 20 mg/ml (Roche #03115879001) was added to each sample and 10 μl to each input and incubated for 1-2 h at 55°C.

#### Phenol/chloroform extraction

Two-milliliter Phase Lock Gel™ Heavy tubes (Fisher #FP2302830) were spinned at RT for 30 s at maxG to pellet gel. In the fume hood, samples were aliquoted into phase lock tubes and an equal volume (∼200 μl) of phenol/chloroform/isoamyl alcohol was added (Sigma #77617), mixed well and spinned at RT for 5 min at maxG. The aqueous phase (aprox 200μl) was transferred into new LoBind 1.5 ml tubes.

#### Ethanol precipitation

Two volumes ethanol 100% (∼400 μl) were added to the previously prepared aqueous solutions. Then, an additional 8-μl 5 M NaCl (final concentration 200 mM NaCl or 1:10 vol 3 M sodium acetate) were added, as well as 1-μl glycogen 20 μg/μl. The samples were mixed well and frozen at -80°C for at least 1 h. Tubes were then spinned in a bench-top microfuge at top speed for 30 min at 4°C, washed with 1 ml of cold 70% ethanol solution, and spinned again at full speed for 10 min at 4°C. The supernatant was carefully removed, and wash step was repeated. The supernatant was removed again, and the pellet was dried in a Speedvac. DNA was resuspended in 30 μl of 10 mM Tris-Cl, pH 8.1.

#### ChIP-qPCR

A mix of the adequate PCR primers (5 mM each) was prepared. Primers were designed to amplify 50- to 150-bp fragments under very stringent conditions (i.e., Tm 58-60°C) and were tested both *in silico* and empirically for little or no unspecific amplification. The qPCR mixes were prepared containing: 14 μl of H_2_O; 4 μl of 5× PyroTaq EvaGreen qPCR Mix Plus (CMB Cultek Molecular Bioline #87H24-001), and 1 μl of isolated DNA. A plate containing 1 μl of primer mix and 19 μl of qPCR mix was prepared, and RT-qPCR was performed using an Applied Biosystems 7300 Real-Time PCR System thermocycler with the following protocol: initial denaturation 95°C for 15 min; then 40 cycles of denaturation 95°C for 15 s, annealing 60°C for 29 s and elongation 72°C for 29 s.

#### List of ChIP-qPCR primers

GAPDH promoter forward, TTCACCTGGCACTGCACAA;

GAPDH promoter reverse, CCACCATCCGGGTTCCTATAA;

GAPDH gene forward, CTACCCAAAAGGGACACCTACAA;

GAPDH gene reverse, TTTCTTATCTTACCCTGCCATGAG;

Arc promoter forward, GCATAAATAGCCGCTGGTGG;

Arc promoter reverse, GAGAACTCGCTTGAGCTCTGC;

Arc gene forward, TCTCCAGGGTCTCCCTAGTC;

Arc gene reverse, CCCATACTCATTTGGCTGGC;

Fos promoter forward, GCAGTCGCGGTTGGAGTAGT;

Fos promoter reverse, CGCCCAGTGACGTAGGAAGT;

Fos gene forward, GCTTCCCAGAGGAGATGTCTGT;

Fos gene reverse, GCAGACCTCCAGTCAAATCCA;

Tubb5 promoter forward, GCCTCTTCTGCCTCTTAGAACCTT;

Tubb5 promoter reverse, TCTGGGCCGGTCTCAGACT;

Tubb5 gene forward, AGCGAACGGAGTCCATAGTC;

Tubb5 gene reverse, CAGGTGGCAAGTATGTCCCT.

### Data analysis

Western blotting fold change data and ChIP-qPCR % of input data were generated from four to seven animals per group (control or trained). For Western blot analysis, three to five replicate wells in independent gel runs were used per animal, with seven animals per group (control or trained); and a minimum of four to six animals per group (control or trained) for ChIP-qPCR analysis (with a minimum of two replicate C_T_ measurement repeats per qPCR experiment). Data were expressed as mean ± SEM and statistically evaluated at a significance level of 5% with unpaired Student’s *t* test (*p < 0.05; comparing control to trained groups for the Western blot analysis; or control to trained groups, and Ser5 to Ser2 levels, for the ChIP-qPCR analysis for each individual target, i.e., promoter or gene body) or two-way ANOVA, using GraphPad Prism (GraphPad Software). Results were represented as mean ± SEM. For behavioral analysis, a one-way ANOVA was used to evaluate acquisition of lever pressing, distances to target and percentage of end-target hits. Statistical significance was set at α = 0.05. Figure symbols are as follows: *p < 0.05, **p < 0.01, ***p < 0.005; n.s., *p* > 0.05.

## Results

### Mice gradually shape their behavior in a fast lever-pressing task

**Figure 1.**
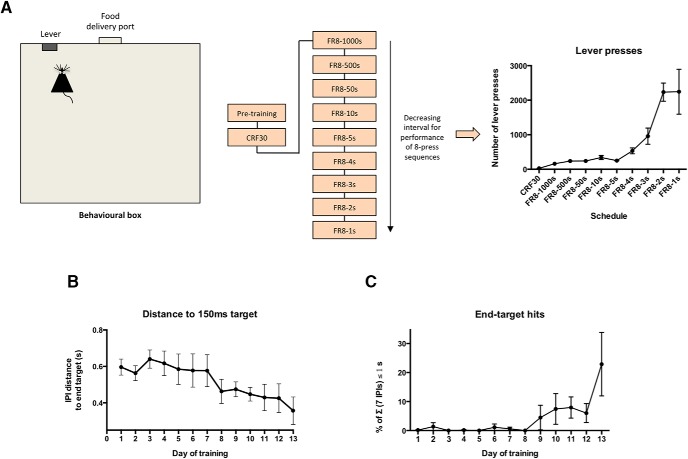
Animal performance during a fast lever-pressing task. After one session of continuous reinforcement with self-paced delivery of up to 30 food pellets (CRF30), animals (*n* = 7) were required to perform on a FR schedule, whereby eight lever presses resulted in delivery of a food pellet within a time contingency, which ranged from one-thousand to 1 s (FR8-1000s to FR8-1s). ***A***, Scheme representing the behavioral setup and structure of the fast lever-pressing task, as well as the task acquisition as represented by the average number of lever presses for each day of training (*F*_(9,52)_ = 22.59, *p* = 0.0009). ***B***, Distance of all seven consecutive IPIs from the final covert target (*F*_(2.155,12.93)_ = 4.638, *p* = 0.0283). ***C***, Percentage of sequences containing the minimum frequency target of the last session (end-target: 7 IPIs < 1s, ∼8.0 Hz; *F*_(12,91)_ = 2.765, *p* = 0.0030); mean ± SEM represented in all graphs.

To examine the impact of learning a motor skill on RNA Pol II RPB1 phosphorylation dynamics, we trained animals in a fast lever-pressing operant task. In this task, animals were first taught to relate pressing a lever with receiving a food pellet in a continuous reinforcement schedule (CRF), with one lever press resulting in delivery of one food pellet to the magazine, to a maximum of 30 pellets per session. After CRF, animals were asked to perform eight lever presses to receive one food pellet (i.e., with a FR of eight lever presses per food pellet; FR8), but having to do so within a time limit that gradually became shorter: FR8-1000s (i.e., eight lever presses within 1000 s), FR8-500s, FR8-50s, FR8-10s, FR8-5s, FR8-4s, FR8-3s, FR8-2s, and FR8-1s, with animals finishing their fast lever-pressing training pressing the lever at 8 Hz (Fig. 1*A*).

Mice showed an increase in the average number of lever presses per session (Fig. 1*A*). This tendency for an escalation in lever pressing is explained by the increasing difficulty in the training regime, as sessions progress toward decreasing time limits in which to perform the sequences of eight lever presses. An analysis of sequence performance across training demonstrates that mice displayed gradually decreasing distances to the final target of 150 ms (as the optimized IPI at FR8-1s: 7 IPIs of ∼150 ms each; Fig. 1*B*), and an increasing percentage of press bouts that would correspond to the target frequency of the last session (end-target: 7 IPIs < 1s; Fig. 1*C*). These data indicate that mice learned to perform this motor skill, which is dependent on striatal plasticity (Jin and Costa, 2010; Jin et al., 2014; Santos et al., 2015).

### Motor skill learning modulates RNA Pol II RPB1 phosphorylation in the striatum

**Figure 2.**
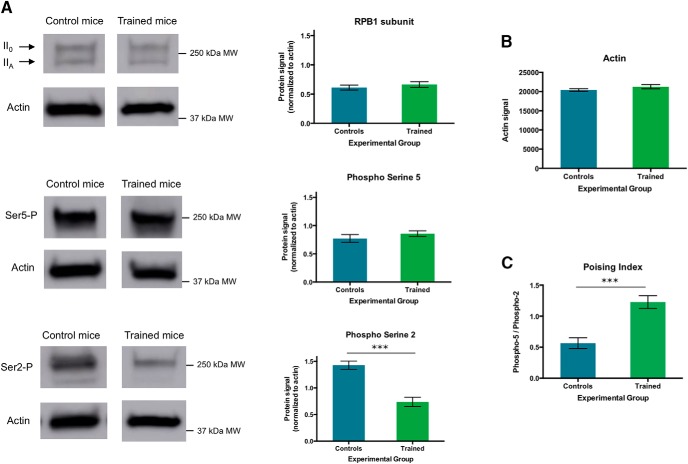
RNA polymerase II RPB1 phosphorylation in the striatum of mice trained in a fast lever-pressing task. ***A***, Immunoblot analysis of the total RPB1 CTD repeat YS_2_PTS_5_PS_7_, with indication of its hyperphosphorylated (II_0_) and hypophosphorylated (II_A_) forms, Ser5P^+^-enriched RPB1 CTD and Ser2P^+^-enriched RPB1 CTD. ***B***, Actin quantification across both phospho-isoforms. ***C***, RNA polymerase II poising index (calculated as the quotient between the Ser5-P and Ser2-P RPB1 CTD phospho-isoforms) in the striatum of mice trained in the fast lever pressing. For both control and trained groups, *n* = 7; data as mean ± SEM; ***p < 0.005.

To test whether motor skill learning had an impact on striatal levels of RNA Pol II RPB1 CTD phosphorylation, we assayed total protein extracts from the striatum of mice trained in the fast lever-pressing task, as well as from control mice, with an antibody that recognizes total RPB1 CTD regardless of the specific phosphorylated residues (Fig. 2).

Because of the varying degrees in residue phosphorylation, protein extracts probed with an anti-RPB1 CTD antibody resolve in two different bands around 250 kDa: that corresponding to the hyperphosphorylated (II_0_), and hence heavier, form of the RPB1 CTD, and the lighter hypophosphorylated (II_A_) form. As expected, we observed no significant differences in the global levels of RPB1 CTD across trained and control mice (Fig. 2*A*). This is not surprising, as what was anticipated were learning-induced substantial differences in the phosphorylation levels within the pool of existing RNA Pol II molecules, and not a bulk change in the number of total RNA Pol II molecules. As RNA Pol II molecules elongate toward productive transcription, the balance between Ser5P^+^- and Ser2P^+^-enriched RPB1 CTD changes: as RNA Pol II is released from the promoter-proximal paused state by the P-TEFb complex, the RPB1 CTD increases the levels of phosphorylation of Ser2 in the RPB1 CTD (Jonkers and Lis, 2015). In other words, by phosphorylation of RPB1 on Ser2, Ser5P^+^ RNA Pol II molecules overcome transcriptional poising and transition to the actively transcribing, elongating form of RNA Pol II. The relation between promoter-rich Ser5P^+^ RNA Pol II and elongating Ser2P^+^ RNA Pol II is what is known as the poising index, which provides a readout of the relationship between these two phosphorylation forms and the rough transcriptional phase RNA Pol II molecules occupy (Jonkers and Lis, 2015). Therefore, we asked whether we would observe a modulation of the phosphorylation levels of RNA Pol II RPB1 CTD at specific serine residues as a result of mice undergoing the motor skill-learning paradigm. We did not observe a significant difference in the levels of RPB1 Ser5P^+^-enriched CTD between control and trained mice (Fig. 2*A*). However, when we examined the levels of Ser2P^+^-enriched CTD, we observed a marked decrease of signal in trained animals when compared with controls (Fig. 2*A*). To rule out the possibility of the phosphorylation differences found between trained and control (or context control) animals being exclusively due to the absence of the “reward” food pellets received by trained mice during sessions, and not to learning of the motor skill itself, we also compared the levels of Ser5P^+^- and Ser2P^+^-enriched RPB1 CTD between context control and reinforcement control animals (mice which were exposed to the same behavioral boxes as trained mice but received ∼30 pellets as a result of the exposure session so as to mimic a food pellet reward similar to that received by trained animals), finding no significant differences between these two groups for either phosphorylation form (*p* = 0.2576 and *p* = 0.0963 for Ser5P^+^-RPB1 CTD and Ser2P^+^-RPB1 CTD, respectively). To test whether these differences in RPB1 phosphorylation were due to fluctuations in the global transcriptional levels in the striatum as a result of training, we compared the actin levels between control and trained mice but found no statistically significant differences between them (Fig. 2*B*).

The decrease in levels of Ser2P^+^-enriched CTD suggest an increase in RNA Pol II poising after learning. To examine this more directly, we calculated a poising index as the ratio between the mainly promoter-bound Ser5P^+^ RPB1 and the actively transcribing Ser2P^+^ RPB1, providing an indication of the balance between these two phosphorylation forms. As expected from the decreased Ser2P^+^ signal, we observed a robust difference between trained animals and control mice, with a significant increase in the poising index of RNA Pol II in the striatum of trained animals (Fig. 2*C*).

### Learning a motor skill modulates RNA Pol II poising at IEGs in the striatum

A previous study has shown neuronal activity-regulated modulation of RNA Pol II poising in *in vitro* cortical cultures in an activity-dependent manner (Saha et al., 2011). This study also showed that priming of IEGs [genes that are rapidly and transiently activated in response to neuronal activity, such as *Arc* (Lyford et al., 1995) and *Fos* (Dragunow and Robertson, 1987)] by poised RNA Pol II was, at least partly, responsible for their fast induction kinetics on neuronal activity. It has also been shown that learning a motor skill, either performing a rotarod task or a skilled-reaching paradigm, modulates the levels of *Arc* and *Fos* in the striatum *in vivo*, demonstrating a learning-dependent modulation of IEG expression in this brain structure (Bureau et al., 2010; Qian et al., 2015). We therefore investigated whether the training-induced modulation of RNA Pol II CTD phosphorylation was observed at IEGs. We performed ChIP followed by quantitative real-time PCR (ChIP-qPCR) on whole striata dissected from control mice and mice trained in the lever-pressing task presented above (Fig. 1*A*).

**Figure 3.**
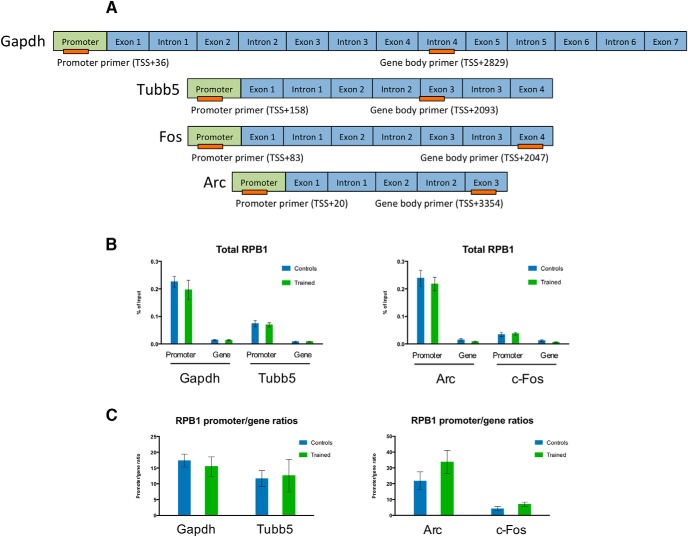
Enrichment of RNA polymerase II RPB1 CTD phosphorylation forms at IEGs in the striatum of mice trained in a fast lever-pressing task. ***A***, Graphical representation (not to scale) of the relative position of primers used in ChIP-qPCR experiments (primers represented in orange). ***B***, ChIP-qPCR analysis of total RPB1 CTD binding at *Gapdh* and *Tubb5* (positive control targets) and *Arc* and *Fos* (IEGs); controls *n* = 5; trained *n* = 6. ***C***, ChIP-qPCR % of input data as a ratio between the promoter and gene bodies of all genomic targets for the total RPB1 CTD repeat. Data as mean ± SEM.

As expected, when we examined total RNA Pol II binding (regardless of phosphorylation) to the promoters and gene bodies of *Arc* and *Fos* (the most common IEGs), and *Gapdh* and *Tubb5* (positive controls that are supposed to be actively transcribed at all times; Fig. 3*A*), we found no statistically significant binding differences between control and trained mice for any of the promoter or gene targets (Fig. 3*B*). We also analyzed the relation between total RPB1-binding to the promoters and gene bodies of each target in control and trained mice. We found no statistically significant differences between control and trained total RPB1 promoter/gene binding ratios in individual targets (Fig. 3*C*).

**Figure 4.**
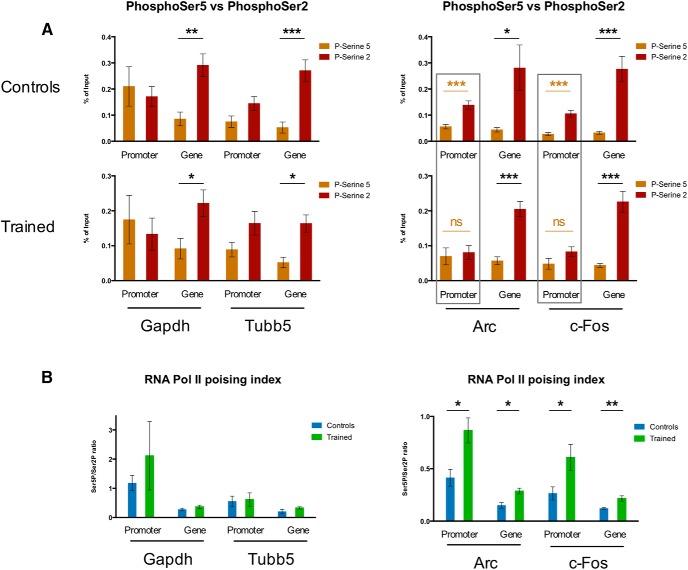
Dynamics of Ser5P^+^- and Ser2P^+^-RPB1 CTD enrichment and resulting poising indices at IEGs in the striatum of mice trained in a fast lever-pressing task. ChIP-qPCR % of input data for Ser5P^+^-enriched RPB1 CTD and Ser2P^+^-enriched RPB1 CTD (***A***) and Ser5P/Ser2P RNA polymerase II RPB1 CTD ratios (poising indices) (***B***) at *Gapdh* and *Tubb5* (positive control targets) and *Arc* and *Fos* (IEGs) in the striatum of mice trained in the fast lever-pressing task (controls *n* = 3; trained *n* = 4). Data as mean ± SEM; *p < 0.05; **p < 0.01; ***p < 0.005; n.s., *p* > 0.05.

We subsequently compared the Ser5P^+^- and Ser2P^+^-RPB1 levels in control and trained mice for all target genes (Fig. 4). We observed a clear pattern of Ser5P^+^- and Ser2P^+^-RPB1 equilibrium with training at the *Arc* and *Fos* IEG promoters, a difference that disappeared completely with training (Fig. 4*A*). This evening out of Ser5P^+^- and Ser2P^+^-RPB1 levels seems to be reversed at the gene body of *Arc* (for *Fos* it seems to be at least maintained). This training-induced modulation of Ser5P^+^- and Ser2P^+^-RPB1-binding does not appear with a comparable extent in the positive control targets, be it promoter or gene body, as the balance between Ser2P and Ser5P in these control and trained groups remains generally stable (Fig. 4*A*).

Next, we examined the poising index (i.e., the Ser5P/Ser2P binding ratios) for the different target genes. We observed an increase in the poising indices for the promoters and gene bodies of both IEGs *Arc* and *Fos* (Fig. 4*B*), consistent with a modulation of the phosphorylation statuses of RNA Pol II molecules bound to these activity-dependent genes. This was not observed in control *Gapdh* and *Tubb5* genes (Fig. 4*B*).

**Figure 5.**
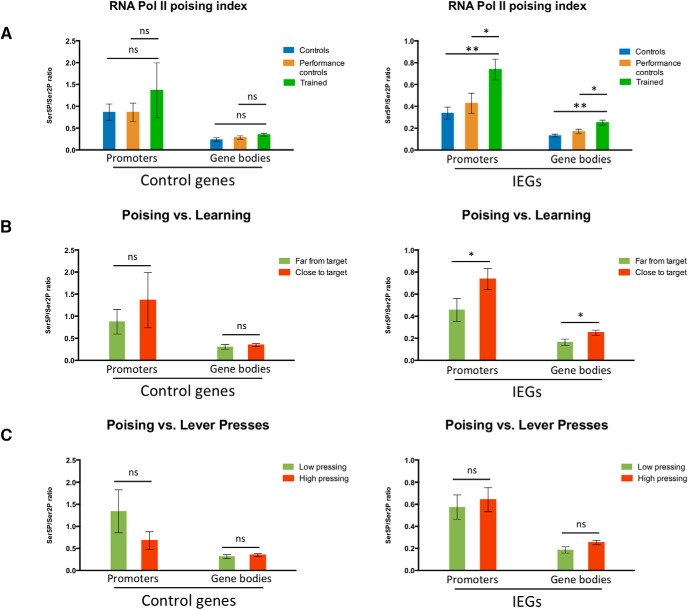
RPB1 CTD poising index at IEGs in the striatum and its correlation with learning of a fast lever-pressing task. ChIP-qPCR % of input data for pooled Ser5P/Ser2P RNA polymerase II RPB1 CTD ratios (poising indices) at the promoters and gene bodies of positive control targets (*Gapdh* and *Tubb5*) and IEGs (*Arc* and *Fos*) in the striatum of (***A***) control and performance control mice (performance control animals were killed after completion of FR8-50s, roughly corresponding to a halfway point in the training regime), as well as mice fully trained in the fast lever-pressing task (controls *n* = 4; performance controls *n* = 4; trained *n* = 3); (***B***) performance control and fully trained mice grouped as, respectively, far from target and close to target, according to the distance to target value (i.e., distance of all seven consecutive IPIs from the final covert target) each animal presented at completion of FR8-50s or FR8-1s schedules, with far from target animals displaying a distance to target value over 0.6 and close to target animals a distance to target value under 0.6; and (***C***) mice trained in the fast lever-pressing task grouped according to the number of lever presses performed in their final training session, with low pressing animals finishing with under 250 lever presses and high pressing animals with over 250 lever presses. Data as mean ± SEM; *p < 0.05; **p < 0.01; n.s., *p* > 0.05.

To guarantee that the observed RNA Pol II phosphorylation modulation resulted from changes associated to learning and not merely triggered by the movement of animals in the operant box, we analyzed the poising index for the different target genes in the striatum of performance control animals (i.e., animals that performed the task extensively but were killed after completion of FR8-50s, before significant learning of the skill; Fig. 1*A*,*C*). We observed no significant difference between the poising indices of these performance control animals, untrained control animals, and trained animals (Fig. 5*A*) for the grouped promoters and gene bodies of control genes. However, we did observe marked differences between trained animals and either control group for the promoters and gene bodies of IEGs (with no significant differences between the poising indices of IEGs in control and performance control animals), indicating that changes in RNA Pol II poising were only observed in animals that learned the skill. Performance control animals were killed immediately after lever pressing training, similarly to control and trained groups, indicating that the differences observed in the poising indices between trained and control mice were not merely due only to ongoing behavior, but related to learning.

To further ensure that the observed differences in Ser5P^+^- and Ser2P^+^-RPB1 phosphorylation levels and resulting poising indices (Fig. 4*A*,*B*) were related to learning of the motor skill, and not performance, we pooled the animals from the performance and trained groups, and segregated them in two halves based on learning (distance to target) or performance (number of lever presses) on the last session. When we segregated animals in groups according to the distance to target value each animal presented (with “far from target” animals displaying a distance to target value over 0.6, and “close to target” animals a distance to target value under 0.6; Fig. 5*B*), we observed no significant differences in the poising indices of the promoters and gene bodies of control genes (*Gapdh* and *Tubb5*) between groups but did note a significant increase in the poising indices of the promoters and gene bodies of IEGs (*Arc* and *Fos*) in animals that were closer to the target value, indicating a correlation between learning and RNA Pol II poising is indeed present. When we analyzed the poising indices of the promoters and gene bodies of control genes and IEGs in mice grouped according to the number of lever presses performed in their final training session (with “low pressing” animals finishing with under 250 lever presses and “high pressing” animals with over 250 lever presses), we found no differences between the groups (Fig. 5*C*), indicating that the observed modulations in RNA Pol II poising levels do not only result from extensive levels of activity, i.e., lever pressing, but from learning to perform the skill.

## Discussion

In this study, we show that learning a motor skill modulates the phosphorylation balance of RNA Pol II RPB1 in the striatum. This molecular regulation occurs at IEGs and suggests a link between learning a striatal plasticity-dependent skill and modulating RNA Pol II poising.

Here, mice learned to perform a motor task in which they were asked to press a lever up to 8 Hz to receive a food reward. Subsequently, total protein from the striata of mice trained in the lever-pressing task was probed with antibodies recognizing the RPB1 CTD regardless of phosphorylation status, as well as Ser5P^+^- or Ser2P^+^-enriched RPB1 CTD. Here, we made two main observations. First, we found no differences in the total levels of RPB1 CTD between control and trained mice. This is not unexpected, given that modulation of RNA Pol II poising-regulated transcriptional programs would more likely involve a dynamic shift in the balance of the specific RPB1 CTD residues being phosphorylated (i.e., a modulation in Ser5P^+^- or Ser2P^+^-enriched RPB1 CTD levels), rather than a massive change in global RNA Pol II binding levels or in the concentration of RNA Pol II molecules in neurons. Secondly, we observed constant levels of Ser5P^+^-enriched RPB1 CTD between control and trained mice, but when we probed total striatal protein for Ser2P^+^-enriched RPB1 CTD, we found a very robust decrease of RPB1 rich in this phosphorylated serine residue. RNA Pol II transitions between RPB1 CTD Ser5 and Ser2 phosphorylation depending on its genomic location, with the most significant peaks for each of these two phosphorylation marks located, respectively, at the promoter or gene body (Peterlin and Price, 2006; Adelman and Lis, 2012; Jonkers and Lis, 2015). However, the levels of Ser5 phosphorylation are maintained to a slighter degree beyond and downstream of gene promoters, as the RPB1 CTD is phosphorylated by P-TEFb on Ser2 and both phosphorylation marks coexists within the same CTD (as Ser5P^+^-RPB1 in RNA Pol II molecules overcome transcriptional poising and transition to actively transcribing RNA Pol II, the elongation form of which is then characterized mainly by Ser2P^+^-RPB1; Peterlin and Price, 2006; Adelman and Lis, 2012; Jonkers and Lis, 2015). For this reason, relatively constant levels of Ser5P^+^-RPB1 CTD concomitant with a decrease in Ser2P^+^-RPB1 levels would be consistent with a shift from actively transcribing to promoter-poising RNA Pol II as a response to neuronal activity and equally indicate poising modulation, a shift that could constitute a true molecular hallmark of learning. In agreement with this hypothesis, and as a consequence of the difference in Ser2P^+^ RPB1 phosphorylation, the poising index for trained mice is remarkably higher than that of controls.

When we then examined the presence of RPB1 at IEGs, we found an overall modulation of RNA Pol II binding toward the promoters of these genes concomitant with the global decrease in Ser2P^+^-enriched RPB1 observed at the protein level. This modulation seems to be learning-specific, as it is not observed in animals with less training, nor in animals with high number of presses that did not become better at fast sequences of pressing. This shift of poising indices at IEG promoters is consistent with the previously suggested role for RNA Pol II poising in conferring a kinetic advantage to the transcription of rapidly induced IEGs, such as *Arc* and *Fos* (Saha et al., 2011; Saha and Dudek, 2013), as well as changes in expression of Arc and C-fos in striatum after skill learning (Bureau et al., 2010; Qian et al., 2015). The onset of neuronal activity had been shown previously as capable of inducing PTEF-b recruitment to IEGs, promoting the subsequent activity-dependent phosphorylation of RPB1 at Ser2 of its CTD, releasing RNA Pol II molecules from a promoter-bound state and allowing them to transition to active elongation (Saha et al., 2011). A shift toward increasing poising indices in mice subjected to a learning paradigm was also observed at IEGs in our ChIP experiments, suggesting a possible role for RNA Pol II poising in learning consolidation, by fine-tuning gene responses to consistent neuronal activity in a precisely timed manner. RNA Pol II poising might also be responsible for maintaining an active transcriptional state at specific genomic loci, as knock down of negative elongation factor, one of the main actors in RNA Pol II poising, results in nucleosome reoccupation of previously nucleosome-free promoter regions, thus hindering transcription factor access to promoter, and promoter proximal, *cis*-regulatory elements (Gilchrist et al., 2010). In this study, we used IEGs as a proof of concept, but the observed modulation of RNA Pol II poising will very likely be differentially expressed in diverse neural circuits and present in different genes in various cell types, as responses to different instances of learning will be sustained by different neural systems.

RNA Pol II poising may be involved in learning at different levels, from allowing for faster transcription in circuits previously activated and involved in learning, to facilitating further learning via use of the same circuits/cells previously involved (Won and Silva, 2008; Silva et al., 2009; Zhou et al., 2009). It has been previously shown that neurons that are molecularly primed are more likely to be involved in learning new memories, or in shaping/reconsolidating existing memories (Won and Silva, 2008; Silva et al., 2009; Zhou et al., 2009; Lopez de Armentia et al., 2007). Our working hypothesis is that activity-dependent modulation of RNA Pol II poising at specific neural plasticity loci during learning will result in long-lasting changes in genomic access and speed of transcription (i.e., the accessibility of specific genomic loci to transcriptional regulatory factors) that will prime the neurons involved in the memory for further learning or consolidation, conceptually extending Waddington’s epigenetic landscape to a neuronal chromatin map, where primed genomic regions in specific neurons will result in primed neurons/circuits. Therefore, priming and faster transcription of IEGs may render neurons, where these genes are poised, more likely to participate in further learning.

In conclusion, we show that learning a motor skill impacts on the *in vivo* striatal balance of RNA Pol II poising, resulting in an increase in the RPB1 poising index in trained mice. We demonstrate the presence of this learning-dependent modulation at the IEGs *Arc* and *Fos*, supporting a new instance of transcriptional modulation induced by learning in the adult brain. Further studies bringing together circuit-specific molecular profiling with the investigation of activity-dependent neuronal transcription should prove a fruitful ground for future research.

## Synthesis

Reviewing Editor: Christian Lüscher, University of Geneva Decisions are customarily a result of the Reviewing Editor and the peer reviewers coming together and discussing their recommendations until a consensus is reached. When revisions are invited, a fact-based synthesis statement explaining their decision and outlining what is needed to prepare a revision will be listed below. Note: If this manuscript was transferred from JNeurosci and a decision was made to accept the manuscript without peer review, a brief statement to this effect will instead be what is listed below.

The authors may want to explicitely define “RNA Pol poising” when first used the abstract.

Other than that I am happy with the responses to the referees that were reviewing the paper for J Neurosci.
